# Die segmentale und somatische Dysfunktion: Wie funktioniert Manuelle Medizin?

**DOI:** 10.1007/s00132-022-04230-z

**Published:** 2022-03-08

**Authors:** Hein Schnell, Florian Max-Josef Wagner, Hermann Locher

**Affiliations:** 1 Praxis für Orthopädie und Unfallchirurgie, Baldestr. 8, 80469 München, Deutschland; 2 Starnberg, Deutschland; 3 Zentrum für Orthopädie und Unfallchirurgie, Tettnang, Deutschland

**Keywords:** Schmerz, Osteopathie, Propriozeption, Manipulation, Blockierung, Pain, Osteopathy, Proprioception, Manipulation, Segmental reflex

## Abstract

**Hintergrund:**

Manuelle Medizin basiert auf neurophysiologischen und biomechanischen Zusammenhängen. Gestörte sensomotorische Regulation führt zu segmentalen und somatischen Dysfunktionen. Auf spinaler Ebene entstehen über die segmentale Zuordnung somatosensorische und vegetative Fehlleistungen. Klinisch imponieren lokale Schmerzen, „Referred-pain“-Syndrome sowie diffuse viszerale Beschwerden. Über myofasziale Strukturen können diese Fehlfunktionen in andere Körperregionen übertragen werden und von dort wiederum über die segmentalen neuronalen Verschaltungen weitere Folgeerscheinungen nach sich ziehen. Die Manuelle Medizin widmet sich diesen Zusammenhängen.

**Techniken:**

Der therapeutische Erfolg manueller Interventionen entsteht durch Einflussnahme auf das propriozeptive System, wodurch die sensomotorische Regulation verbessert werden kann. Propriozeptive Reize wirken zudem direkt schmerzhemmend im Zentralnervensystem (ZNS). Manuelle Techniken können primär Gelenke, myofasziale Strukturen und auch die Viszera zum Zielorgan haben, gemeinsames Ziel ist das Setzen von propriozeptiven Reizen mit gezielter segmentaler Zuordnung.

**Pathologie:**

Das Verständnis der neurophysiologischen und biomechanischen Zusammenhänge kann ein wertvolles differenzialdiagnostisches Instrument sein, insbesondere bei scheinbar inkonsistenter Befundkonstellation. Auch strukturelle Pathologien können reflektorisch zu Dysfunktionen führen. Ob die strukturelle Pathologie oder die Dysfunktion führend für das klinische Bild verantwortlich ist, lässt sich häufig durch eine probatorische manuelle Therapie beurteilen. Lege artis indiziert und durchgeführt besteht ein exzellentes Nutzen-Risiko-Verhältnis.

## Abstract (en)

**Background:**

Manual medicine is based on neurophysiologic and biomechanical principles. Impaired senso-motor regulation causes segmental and somatic dysfunction. Via segmental mapping, somato-sensory and vegetative dysfunction arises. Local pain, referred pain, and vague symptoms can occur in the thoracic and abdominal regions. Myofascial structures can transfer these dysfunctions to other body areas, where segmental neuronal connections lead to further dysfunction. Manual medicine addresses these aspects.

**Techniques:**

Manual interventions work by influencing the proprioceptive system, improving senso-motor regulation. Furthermore, proprioceptive stimulation inhibits pain mechanisms in the CNS (spinal and cerebral). Manual techniques may focus on joints, myofascial, or visceral structures. The main common target is to induct proprioceptive stimulus into related spinal segments.

**Pathology:**

Knowledge of these neurophysiologic and biomechanical aspects can be a powerful tool in differential diagnostics, particularly in cases of apparently inconsistent clinical findings. Additionally, structural pathology can lead to reflex-mediated dysfunction. To determine whether structural pathology or functional derangement is accountable for symptomatology, a manual approach on a trial basis can be very helpful. Lege artis indicated and performed, manual medicine offers an excellent risk–benefit ratio.

Manuelle Medizin wird alltäglich in der Primärversorgung mit sehr guten Ergebnissen angewandt. Teilweise wird die Methode auch kritisch gesehen. Die segmentale und somatische Dysfunktion ist die Diagnose, auf deren Boden die Indikation zur manualmedizinischen Intervention gestellt wird. Dieser Übersichtsartikel stellt die grundlegenden neurophysiologischen und biomechanischen Mechanismen vor. Es wird gezeigt, wie diese Mechanismen zu einer palpatorisch fassbaren Pathologie (segmentale und somatische Dysfunktion) führen und warum manuelle Behandlung diese Pathologie wieder auflösen kann. Die weiteren Artikel dieses Themenhefts bauen auf diesen neurophysiologischen Grundlagen auf.

## Einleitung

Manuelle Medizin wird täglich tausendfach durchgeführt und von Patienten wegen der teils frappierenden Erfolge häufig nachgefragt. Anlass sind meist lokale und/oder ausstrahlende („referred pain“) Schmerzen an der Wirbelsäule, aber auch Symptome wie Schwindel (besser: Dizziness), Tinnitus oder abdominelle Beschwerden können von einer manuellen Intervention profitieren. In manchen Fällen dieser Symptome scheint die manuelle Therapie aber zu versagen, weshalb einige Fachgruppen der Manuellen Medizin grundsätzlich kritisch gegenüberstehen.

Damit eine manuelle Therapie zu einer Reduktion der Beschwerden führt, muss eine adäquate manualmedizinische Diagnose vorliegen, denn behandelt wird nicht ein Symptom, sondern immer eine sogenannte Dysfunktion [4]. Das heißt, die Beschwerden müssen Folge einer Dysfunktion sein. Auflösen der Dysfunktion bessert dann mittelbar die Symptome. Beispielsweise wäre eine Behandlung an der Halswirbelsäule bei Drehschwindel wegen Kreislaufschwäche bei AV-Block grundlegend fehlindiziert und kann selbstredend nicht von Erfolg gekrönt sein. Liegt aber bei leichtem ungerichtetem Schwindel (besser: Dizziness) eine HWK-2-Dysfunktion vor, so kann deren Behandlung wegweisend sein.

Dieser Artikel befasst sich mit der segmentalen und somatischen Dysfunktion sowie ihren neurophysiologischen und biomechanischen Grundlagen. Daraus abgeleitet wird dargestellt, wie manualmedizinisch diagnostiziert wird und wie manuelle Interventionen wirken.

## Die segmentale und somatische Dysfunktion (ICD 10: M99.0X) und ihre neurophysiologische Grundlage

Die segmentale und somatische Dysfunktion ist die zentrale Diagnose in der manuellen Medizin. Auf ihr basiert die Indikation zur manualmedizinischen Intervention [4].

Die Diagnose der segmentalen und somatischen Dysfunktion wird klinisch palpatorisch gestellt

Neben Anamnese und allgemeiner klinischer Untersuchung werden in einem klar definierten Algorithmus palpatorische Befunde erhoben, die zu einer manualmedizinischen Diagnosen führen. Diese Diagnose wird synonym im deutschen Sprachraum meist als Blockierung bezeichnet [2, 4].

**Figure 1:**
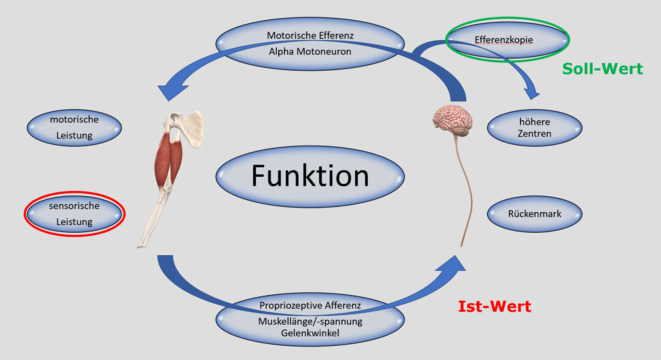

Die physiologische Funktion am Bewegungsapparat basiert auf einem sensomotorischen Regelkreis (Abb. 1). Ist dieser gestört, kann eine Dysfunktion entstehen.

### Sensomotorischer Regelkreis

Für eine Bewegungs- oder Haltearbeit (motorische Leistung) bedarf es eines Befehls (motorische Efferenz) aus höheren Zentren des ZNS (vom Stammhirn bis zum motorischen Kortex). Gleichzeitig findet in den Strukturen des Bewegungsapparates (Muskulatur, Gelenkkapsel, Bandstrukturen etc.) auch eine permanente Sensorik statt (Gelenkwinkel, Muskellänge/-Spannung), die Propriozeption. Diese propriozeptive Information fließt über afferente Bahnen zurück in das ZNS. Hier wird dies als Ist-Wert mit der sog. Efferenzkopie (nach Anochin) als Soll-Wert abgeglichen. Bei Differenzen zwischen Soll- und Ist-Wert muss entsprechend mit neuer motorischer Efferenz korrigiert werden. Zudem dient der neue Ist-Wert wiederum als Basis für den nächsten motorischen Befehl. Es resultiert ein sensomotorischer Regelkreis (abgeleitet aus [1]). Wesentlich ist hierbei, dass ohne korrekte Information über den aktuellen Gelenkwinkel und Muskellänge/-spannung kein gutes Bewegungsprogramm abgerufen werden kann. Analogie: Ein Navigationsgerät ohne GPS-Empfang liefert keine suffiziente Zielführung.

### Konvergenz auf segmentaler Ebene

**Figure 2:**
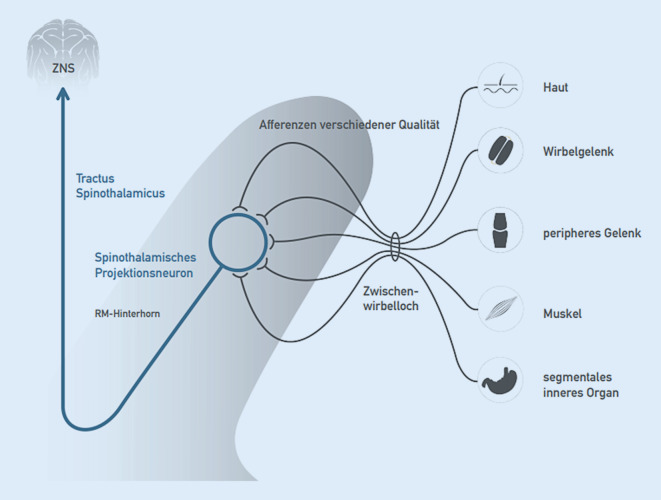

Afferenzen verschiedener Qualitäten aus unterschiedlichen Geweben konvergieren im Hinterhorn auf eine Neuronenpopulation, die vereinfacht als ein Neuron betrachtet wird (Abb. 2). Man spricht vom (multirezeptiven) spinothalamischen Projektionsneuron, oft auch „Wide-dynamic-range“-Neuron genannt [6, 12]. Hier werden alle Informationen, z. B. Temperatur, Druck, Tastsinn, Vibration, Schmerz und Propriozeption (Lagesinn) aus der Peripherie zusammengeführt, verarbeitet und weitergeleitet.

### Noziafferenz und (schutz-)reflektorische motorische Antwort

**Figure 3:**
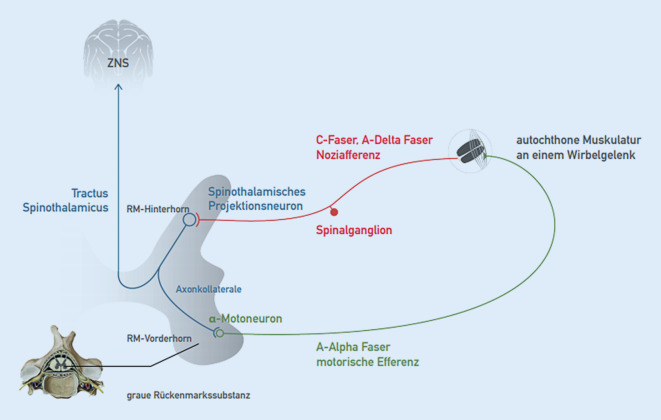

Segmentale Noziafferenzen, z. B. aus Wirbelgelenken, Haut, Muskulatur oder auch inneren Organen werden primär im spinothalamischen Projektionsneuron verarbeitet (Abb. 2). Zum einen folgt die Weiterleitung über den Tractus spinothalamicus nach zentral, wo dann u. a. die topische Zuordnung und bewusste Wahrnehmung stattfinden. Zum anderen kommt es über Alpha-Motoneurone zu einer Aktivierung segmental zugeordneter Muskulatur, zu der auch die segmental innervierte autochthone Rückenmuskulatur zählt (Abb. 3). Die Beweglichkeit im betroffenen Wirbelsäulenabschnitt wird reduziert (blockiert) [5, 12].

Der Prozess, der zu einer segmentalen und somatischen Dysfunktion führt, stimmt prinzipiell mit einem Schutzreflexgeschehen überein. Analog zur Aktivierung der Armbeuger bei Kontakt der Hand mit einer heißen Herdplatte führen segmentale Noziafferenzen zur Aktivierung der autochthonen Rückenmuskulatur im gleichen Segment [5, 12].

Löst sich diese muskuläre Aktivierung nicht wieder und besteht fort, resultieren Fehler im sensomotorischen Regelkreis, was zu einem Circulus vitiosus führen kann. Dauerhaft angespannte Muskulatur kann ihre eigene Länge und Spannung nicht gut messen, diese mangelhafte Propriozeption führt im Weiteren zu mangelhaften motorischen Efferenzen etc.

Zwei Faktoren sind für diese anhaltende Aktivierung ausschlaggebend:

- Ein anhaltender und/oder ausreichend starker nozizeptiver Reiz im betroffenen Segment.
- Ein erhöhter Grundtonus der Muskulatur. Dieser wird über die von Gamma-Motoneuronen innervierten Muskelspindeln reguliert und z. B. durch absteigende Bahnen aus dem limbischen System beeinflusst [4].

Dies ist der grundlegende Pathomechanismus der segmentalen und somatischen Dysfunktion. Dieser Pathomechanismus ist grundsätzlich reversibel, weshalb teilweise auch der Begriff der reversiblen Funktionsstörung verwendet wird.

### Vegetative Innervation und sympathische Systemaktivierung

**Figure 4:**
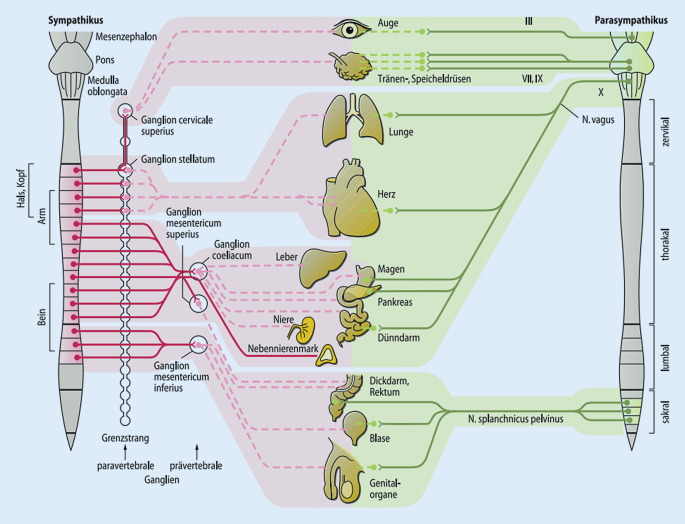

Auch das vegetative Nervensystem ist in den segmentalen Verschaltungen repräsentiert. Es kann wesentlichen Einfluss beim Entstehen einer segmentalen und somatischen Dysfunktion haben. Die sympathischen Ursprungskerne liegen vorwiegend thorakal, die parasympathischen Ursprungskerne sind v. a. im Hirnstamm und im Sakralbereich zu finden (Abb. 4; [16, 17, 20]).

#### Sympathikus

**Figure 5:**
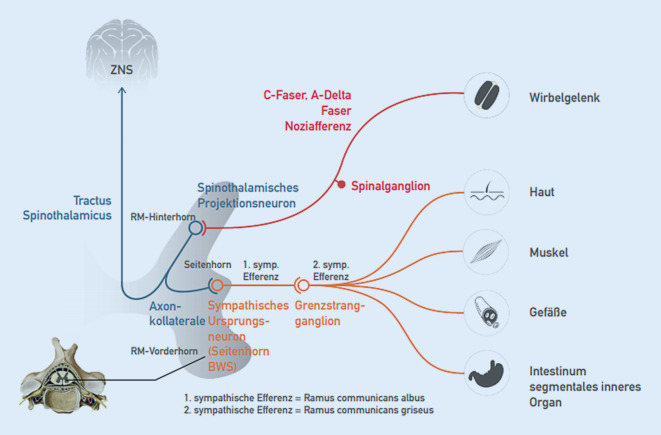

Die sympathischen Efferenzen dienen vor allem der Regulation der Organfunktionen und der Durchblutung. Das sympathische Ursprungsneuron (1. Neuron) liegt im Seitenhorn und erreicht über die Vorderwurzel via Ramus communicans albus den Grenzstrang (Abb. 5). Hier erfolgt teilweise eine Umschaltung auf ein 2. Neuron, teilweise werden die Fasern zu anderen prävertebral und organnah gelegenen Ganglien geleitet. Das 1. Neuron wird über Axonkollateralen ähnlich der Alpha-Motoneurone (Abb. 3) auch vom spinothalamischen Projektionsneuron gespeist (Abb. 5). Eintreffende Noziafferenzen am spinothalamischen Projektionsneuron können folglich auch die sympathische Aktivität modulieren [12, 21]. Das vegetative Nervensystem spielt bei der segmentalen motorischen (Reflex‑)Antwort eine Rolle.

Das vegetative Nervensystem spielt bei der segmentalen motorischen (Reflex‑)Antwort eine Rolle

Die sympathischen Efferenzen haben somit ebenfalls einen segmentalen Ursprung, der größte Anteil im Bereich der BWS (Abb. 4). Die einzelnen Grenzstrangganglien werden jedoch aus mehreren Segmenten gespeist, im klinischen Bild ist deswegen oft kein eindeutiger Segmentbezug mehr erkennbar.

Parallel zu den sympathischen Efferenzen zu den Organen laufen auch Afferenzen, die z. B. an der Schmerzübertragung aus den Eingeweiden beteiligt sind, zurück zum Rückenmark [18].

#### Parasympathikus

Die parasympathischen Verschaltungen haben ihre Ursprungsneurone v. a. im Hirnstamm sowie präsakralen Ganglien (Abb. 4). Die kranialen Anteile folgen den Hirnnerven III, VII, IX und X sowie den Arterien im Kopfbereich. Die Fasern im N. vagus (X) versorgen alle Thorax- und Bauchorgane bis zum Cannon-Böhm-Punkt (linke Kolonflexur). Der sakrale Anteil des Parasympathikus folgt den Vorderwurzeln der Segmente S2–4 und vermischt sich mit den sympathischen Fasern innerhalb der Nn. splanchnici. Er versorgt den Darm aboral des Cannon-Böhm-Punkts. Auch im parasympathischen System gibt es noziafferente Fasern, sie verlaufen mit dem N. vagus bzw. münden in die Spinalganglien von S2–4 [18]. Das vegetative Nervensystem spielt sowohl beim Vermitteln von viszeralen Noziafferenzen sowie bei der segmentalen motorischen (Reflex‑)Antwort eine Rolle.

## Manualmedizinische Diagnostik

Bei einer manualmedizinischen Untersuchung werden viele Einzelbefunde erhoben: Veränderungen der Gewebespannung, verminderte Gewebeverschieblichkeit, druckschmerzhafte Punkte, endgradige Einschränkungen der Beweglichkeit, Veränderungen im Gelenkspiel und Anschlagsgefühl am Ende der ROM. Jeder Einzelbefund ist zunächst für sich alleine genommen wenig bedeutsam. Zusammengenommen können sich Befundkonstellationen ergeben, auf Basis derer die Diagnose einer segmentalen und somatischen Dysfunktion gestellt werden kann [2, 4].

Die Befunde werden rein palpatorisch erhoben und haben kein Korrelat in einer Bildgebung. Die Befunde haben ihren Ursprung in den oben dargestellten neurophysiologischen Zusammenhängen (segmentale Konvergenz, sensomotorischer Regelkreis und motorische Reflexantwort).

In den verschiedenen manualmedizinischen Schulen im deutschen Sprachraum und im internationalen Vergleich mögen durchaus Nuancen und Unterschiede hinsichtlich der Befunderhebung und Befundbeschreibung bestehen, im Kern sind sie sich jedoch sehr ähnlich.

Auf europäischer Ebene konsentiert und definiert ist die MIP-Diagnostik

Auf europäischer Ebene (European Scientific Society of Manual Medicine [ESSOMM]) konsentiert und definiert ist die MIP-Diagnostik (*M*obility, *I*rritation, *P*rovocation) [2, 4, 14] Man versteht hierunter die Befundkonstellation

- einer segmentalen Hypomobilität (Mobility) *und*
- eine Tonuserhöhung der segmental zugeordneten Muskulatur (Irritation) *und*
- ein typisches Verhalten dieser segmental zugeordneten Muskulatur bei passiver Bewegung im betroffenen Segment (Provocation).

Liegen diese drei Befunde gleichzeitig vor, so wird die Diagnose segmentale und somatische Dysfunktion (Blockierung) ICD 10: M99.0X gestellt.

Diese dreiteilige Befundkonstellation resultiert aus den in Abb. 3 dargestellten Zusammenhängen. Durch den beschriebenen Schutzreflexmechanismus kommt es zur segmentalen Aktivierung der autochthonen Muskulatur, hierdurch wird die Beweglichkeit des betroffenen Wirbelsäulensegments reduziert (segmentale Hypomobilität). Diese eingeschränkte Beweglichkeit kann gut palpatorisch erfasst werden. Die betroffene autochthone Rückenmuskulatur selbst ist ebenfalls gut palpatorisch zugänglich. In ihr finden sich druckschmerzhafte Punkte mit erhöhtem Muskeltonus (Irritationspunkte). Wenn nun ein Wirbelsäulensegment passiv bewegt wird (Provokation) und es kommt bei einer Bewegungsrichtung zur Schmerz- und/oder Tonusreduktion am Irritationspunkt, so spricht man von der sogenannten *freien Richtung*. Diese findet sich, da der noziafferente Einstrom am Projektionsneuron durch die veränderte Position nachgelassen hat, wodurch wiederum die Schmerzweiterleitung nach zentral sowie die motorische Antwort reduziert ist. Umgekehrt kommt es in der *gesperrten Richtung *zu einer Tonus- und/oder Schmerzzunahme am Irritationspunkt.

Strukturelle Ursachen für Wirbelsäulenschmerzen wie Fraktur, Spondylodiszitis, aktivierte Osteochondrose etc. zeigen typischerweise *keine* freie Richtung auf. Gleichwohl bedingen diese Diagnosen ebenfalls Noziafferenzen, die auch zu einer reflektorischen Verspannung der autochthonen Muskulatur führen können (Abb. 3 und 6).

**Figure 6:**
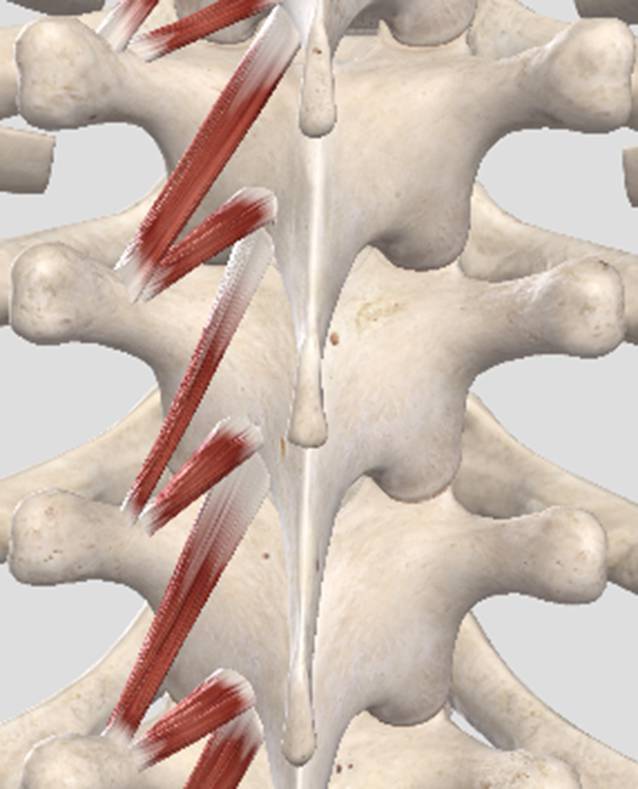

## Manualmedizinische Therapie

### Grundlagen

**Figure 7:**
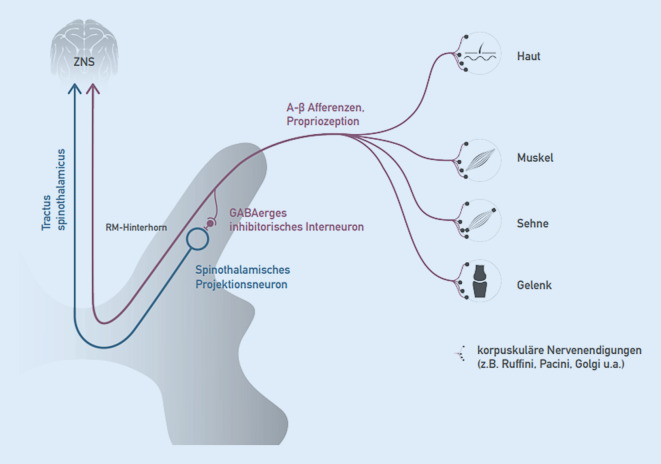

Die Hände des Manualmediziners erreichen die Hautschichten sowie die wesentlichen Strukturen des Bewegungsapparats (Muskeln, Faszien, Gelenke). Diese Strukturen sind besetzt mit Propriozeptoren, die den Lagesinn (Propriozeption) vermitteln. Die Signale der Propriozeptoren werden über A‑Beta-Fasern an oben genanntes spinothalamisches Projektionsneuron geleitet. Die Verschaltung an dieser Stelle erfolgt über inhibitorische GABAerge Interneurone (Abb. 7; [3, 7]).

Gamma-Aminobuttersäure ist einer der wesentlichen inhibitorischen Neurotransmitter im ZNS. Durch diese GABAerge Inhibition kommt es folglich zur Reduktion der Schmerzweiterleitung nach zentral und zu reduzierter (schutz-)reflektorischer motorischer Antwort [3, 7]. Propriozeptive Reize können somit oben beschriebenen Circulus vitiosus durchbrechen (Reversibilität das Pathomechanismus, s. oben). Je genauer das betroffene Segment mit propriozeptiven Reizen erreicht wird, desto besser ist meist der Therapieerfolg. Ob diese propriozeptiven Reize nun aus Gelenkstrukturen, Muskulatur, Faszien oder Haut und Subkutis kommen, ist zunächst zweitrangig. Entscheidend sind vielmehr die Summe der propriozeptiven Reize und ihre segmentale Zuordnung. Darüber hinaus wirkt propriozeptive Stimulation direkt schmerzhemmend im ZNS [13].

### Manualmedizinische Techniken

Die Vorstellung, wie Manuelle Medizin funktioniert, ist nach wie vor stark vom Gelenkbefund geprägt und arbeitet mit einem Gelenkmodell und daraus abgeleitet mit Gelenktechniken. Es mag trivial erscheinen: wird ein Gelenk bewegt, bewegt sich auch die dazugehörige Muskulatur und es ändert sich deren Länge und Spannung. Gelenk und Muskulatur sind untrennbar miteinander gekoppelt.

In den letzten Jahren werden in den Weiterbildungskursen zur Manuellen Medizin zunehmend auch Techniken gelehrt, die auf Dysfunktionen des myofaszialen und viszeralen Systems abzielen [8]. Diese Strukturen können ebenfalls Dysfunktionen (Blockierungen) im neurophysiologischen Sinne aufweisen, die durch gestörte viszerovertebrale und/oder viszerovegetative reflektorische Regelkreise entstehen können.

#### Gelenktechniken

Es wird unterschieden zwischen *Gelenkmobilisation* und *Gelenkmanipulation*. [2, 4]. Es werden Gelenkpartner innerhalb ihres physiologischen Gelenkspielraums gegeneinander bewegt. Bei der Manipulation erfolgt zusätzlich eine kleine schnelle Mikrobewegung (Syn. Impuls) in die freie Richtung. Auch diese Mikrobewegung bleibt streng im physiologischen Gelenkspiel. Vorangeschaltet ist eine Probemobilisation in die freie Richtung, die hinsichtlich Weg und Spannung weit über das hinausgeht, was anschließend als Mikrobewegung sehr schnell durchgeführt wird. Kommt es bei dieser Probemobilisation zu einer Schmerzzunahme oder sonstiger Symptomverstärkung, so darf nicht manipuliert werden!

Manipulative Techniken bedürfen einer ausführlichen Schulung und stringenter kritischer Indikationsstellung. Zwingend müssen gefährliche Kontraindikationen und Differenzialdiagnosen akribisch ausgeschlossen werden. Manipulationen sollten nicht pauschal gleichgesetzt werden mit der paramedizinisch unkritisch angewandten Chiropraktik. Gleichwohl gründet deren eventueller Therapieerfolg ebenfalls auf den genannten neurophysiologischen Zusammenhängen, auch wenn das dem ein oder anderen Therapeuten nicht bewusst sein mag. Keinesfalls sollten Manipulationen als Repositionsmanöver bezeichnet werden, da die hierfür nötige Pathologie einer (Sub‑)Luxation gar nicht vorliegt.

Man geht davon aus, dass Manipulationen einen sehr hohen propriozeptiven Reiz erzeugen, der sehr gezielt im gestörten Segment aufläuft [3, 7].

In der englischsprachigen Literatur werden unter „manipulation“ alle manuellen Techniken verstanden, im deutschen Sprachgebrauch wird allein die oben beschriebene Impulstherapie als Manipulation bezeichnet.

#### Myofasziale Techniken (synonym: Weichteiltechniken)

Es können nicht alle myofaszialen Techniken an dieser Stelle beschrieben werden. Da myofasziale Strukturen gut von den therapeutischen Händen zu erreichen sind und eine hohe Propriozeptorendichte aufweisen (Muskelspindeln, Sehnenspindeln), stellen sie gute Zielorgane für manuelle Therapien dar.

#### Viszerale Techniken

Mobilisieren der viszeralen Organe kann direkt eine Funktionsverbesserung bewirken. Beispiel: Kolonmassage bei Obstipation. Auch die Viszera tragen Propriozeptoren, die mit den Händen stimuliert werden können. Auch die Viszera unterliegen einer segmentalen Zuordnung mit funktionssteuernden Regelkreisen [20] (Abb. 4).

#### Kraniale (kraniosakrale) Techniken

Über die hohe Dichte an Propriozeptoren im Kopf- und Gesichtsbereich sowie sakral besteht erhebliches Potenzial zum Setzen propriozeptiver Reize auf Rückenmarksegmente mit hoher parasympathischer Gangliendichte (Abb. 4).

#### Techniken an Kiefergelenken und Kauapparat

Wie kaum an einer anderen Stelle vermischen sich hier artikuläre, myofasziale und vegetative Aspekte manuellen Handelns. Es bestehen komplexe direkte Verschaltungen zur oberen Halswirbelsäule sowie enge Lagebeziehungen und Funktionszusammenhänge mit etlichen Hirnnerven. Näheres hierzu siehe Artikel zur HWS-Dysfunktion in diesem Heft.

## Komplexe Symptomkonstellationen (Verkettung)

Für das Verständnis komplexer Symptomkonstellationen und für das Verständnis, warum manualmedizinische Interventionen komplexe und weitreichende Effekte haben können, sind ergänzend zu den neurophysiologischen Gesichtspunkten auf segmentaler Ebene zwei weitere Aspekte bedeutsam:

- Alle Strukturen am menschlichen Körper unterliegen einer segmentalen Zuordnung, die segmentale Entstehung im ontogenetischen Sinne findet ihr Abbild in der *Segmentanatomie* [20].
- Über die myofaszialen Zugverspannungen wird das Skelett in seiner dreidimensionalen Form aufgerichtet und gehalten. Die beteiligten Strukturen sind mechanisch gekoppelt und bringen so Spannungsänderungen von einer Etage in eine andere Etage. Gleichzeitig sind diese Strukturen auch immer propriozeptiv aktiv. Das muskuloskelettale dreidimensional aufgespannte System kann näherungsweise auch als tensegrales Konstrukt im Sinne des *Tensegrity-Modells* nach Buckminster Fuller gesehen werden [15].

Segmentanatomie und Tensegrity-Model dienen heute als leistungsfähige Erklärung für das, was in der Manuellen Medizin häufig als Verkettung bezeichnet wird.

### Segmentanatomie

Alle Strukturen am menschlichen Körper unterliegen einer segmentalen Zuordnung [19, 20]. So hat jeder Muskel einen segmentalen Ursprung und Bezug (Myotom). Dies zeigt sich konkret in der jeweiligen Innervation. Beispiel: Musculus latissimus, Innervation Nervus thoracodorsalis, radikulärer Ursprung (= segmentale Zuordnung) C6 bis C8.

Gleiches gilt für Knochen (Sklerotom), Viszera (Viszerotom), Haut (Dermatom). Verbunden sind die unterschiedlichen Strukturen eines Segments über den gemeinsamen Spinalnerven. „Der Spinalnerv definiert das Segment“ (Wancura-Kampik) [20]. Grundsätzlich können nun bei einer segmentalen und somatischen Dysfunktion alle -tome betroffen sein, also schmerzhaft erscheinen und palpierbare Texturveränderungen aufweisen. Die einzelnen Segmentanteile können weit verstreut voneinander liegen. Beispiel s. oben C8 mit Anteil an M. latissimus und M. pectoralis major und der ulnaren Handkante (hier liegen Dermatom, Myotom und Sklerotom von C8 übereinander). Zusätzlich liegt auf Höhe HWK 7/BWK 1 auch noch das Ganglion stellatum, auch dieses hat C8-Spinalnerv-Anbindung und ist für viele Vegetativfunktionen der oberen Extremität, Kopf, Hals, Herz und Lunge verantwortlich.

### Neurophysiologische Verkettung

**Figure 8:**
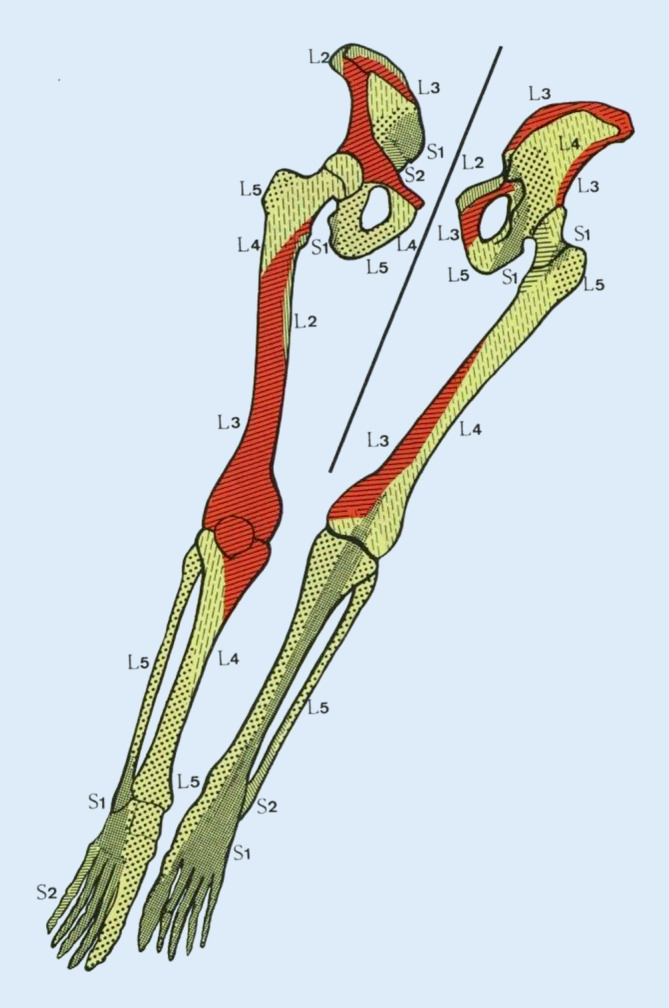

Es resultieren komplexe Wechselbeziehungen zwischen Peripherie und Achsenorgan. Dies gilt sowohl für die Entstehung von Dysfunktionen als auch für die Optionen des therapeutischen Zugriffs über die segmentale Verschaltung. Beispielsweise kann eine Gonarthrose eine niederschwellige Nozizeption erzeugen und zu rezidivierenden LWS-Dysfunktionen führen (Sklerotom L3, Abb. 8).

Gleichzeitig kann die Behandlung (propriozeptive Stimulation) eines Kniegelenks eine schmerzhafte segmentale Dysfunktion in Höhe LWK 3 beeinflussen und umgekehrt sollte bei Knieschmerzen ohne strukturelles Korrelat (z. B. vorderes Knieschmerzsyndrom) auch immer nach einer klinisch stummen LWS-Dysfunktion gefahndet (MIP-Diagnostik) und ggf. therapiert werden.

Eine besondere Bedeutung hat zusätzlich die vegetative Innervation. Über diese lassen sich viele direkte Verbindungen von Wirbelsäulenabschnitten zu inneren Organen erkennen [16, 18, 20, 21]. Hochzervikal und tieflumbal/sakral finden sich viele parasympathische Nervenkerne (Abb. 4). Die meisten sympathischen Ursprungskerne liegen thorakal. Segmentale und somatische Dysfunktionen dieser Wirbelsäulenabschnitte können damit auch die autonomen Funktionen betreffen und es kann zu viszeraler Symptomatik kommen (z. B. funktionelle Magen-Darm-Beschwerden, Reizdarm). Umgekehrt können die inneren Organe auch Ursache einer Wirbelsäulendysfunktion sein (z. B. rezidivierende schmerzhafte BWS-Dysfunktion als erstes Symptom eines Pankreaskarzinoms).

Motorische Fehlleistungen einer Etage können zu sensorischen Fehlleistungen anderer Etagen führen

Für Näheres zu den Auswirkungen auf die inneren Organe siehe auch „Von akutem Koronarsyndrom bis Zoster. Differenzialdiagnostik bei segmentaler und somatischer Dysfunktion an BWS und Rippen“ (H. Schnell 2022) in diesem Heft.

### Mechanisch-strukturelle Verbindungen: Tensegrity-Model

Über die myofaszialen Zugverspannungen werden die Knochen in ihrer dreidimensionalen Form aufgerichtet, gehalten und bewegt. Somit sind alle Wirbelsäulenetagen und die Extremitäten auch direkt mechanisch gekoppelt [15]. Über diese Kopplung können nun auch motorische Fehlleistungen der einen Etage in sensorische Fehlleistungen einer anderen Etage übertragen werden und dort wiederum im Sinne des gestörten sensomotorischen Regelkreis zu einer weiteren motorischen Fehlleistung führen. Dies ist ergänzend zur segmentanatomischen Betrachtung ein weiteres leistungsfähiges Erklärungsmodell dafür, warum Dysfunktionen häufig in Ketten angeordnet sind. Beispiel für eine biomechanische Verkettung: tieflumbale Schmerzen nach OSG-Distorsion: OSG – distales Tibiofibulargelenk – proximales Tibiofibulargelenk – M. biceps femoris – Os Ilium – Iliosakralgelenk.

#### Infobox 1 Das Tensegrity-Model nach Buckminster Fuller (Architekt und Designer 1895–1983)

Tensegrity ist ein Kunstwort aus „tension“ (Zugspannung) und „integrity“ (Ganzheit, Zusammenhalt, Integrität). Es beschreibt Konstruktionen, die aus starren Stäben und Zugverspannungen bestehen. Die Stäbe sind nicht miteinander verbunden, nur die Zugspannung hält die Konstruktion 3‑dimensional aufrecht. Eine Änderung der Spannung an einer Stelle im Konstrukt führt zur Deformation des ganzen Konstrukts und zur Spannungsänderung in den anderen Anteilen. Diese Systeme sind in sich sehr stabil und dynamisch anpassungsfähig. Dieses Modell kann näherungsweise auch auf den menschlichen Körper mit den Knochen (Stäbe) und myofaszialen Strukturen (Verspannungen) angewandt werden [15].

Eine sehr schöne und anschauliche Erklärung von Thomas Myers findet sich als Video unter:

**Video link:** https://www.youtube.com/watch?v=BzgxYpDyO0M **What is Tensegrity, Thomas Myers.**

## Conclusio

Grundsätzlich wird in der Manuellen Medizin nicht ein Symptom, sondern die segmentale und somatische Dysfunktion behandelt [4]. Das Auflösen der Dysfunktion verbessert mittelbar die Symptomatik, sofern die Dysfunktion Ursache der Symptomatik ist. Auch strukturelle Pathologien, z. B. Facettengelenksarthrosen, können als Schmerzgenerator reflektorisch zu Dysfunktionen führen. Ob die strukturelle Pathologie oder die Dysfunktion führend für das klinische Bild verantwortlich ist, lässt sich häufig erst durch Erfolg oder Misserfolg einer manuellen Intervention beurteilen.

Eine Sonderstellung hat das Symptom Schmerz, da manuelle Techniken auch direkt schmerzhemmend wirken. Deshalb findet sich die Manuelle Medizin berechtigterweise auch in den Empfehlungen der *Nationalen Versorgungsleitlinie nichtspezifischer Kreuzschmerz *[9]. Definitionsgemäß liegt hier *keine* Dysfunktion zugrunde. Gleichwohl darf man aus manualmedizinischer Sicht mutmaßen, dass bei sog. unspezifischen Kreuzschmerzen wahrscheinlich häufig die spezifische Diagnose einer segmentalen und somatischen Dysfunktion übersehen, bzw. nicht nach ihr gesucht wurde. Die *S2k-Leitlinie spezifischer Kreuzschmerz* [11] hingegen kennt die Blockierung und die myofasziale Dysfunktion und kommt hier zu einer „Sollte“-Empfehlung für eine manualmedizinische Therapie.

Die Komplexität der neurophysiologischen Zusammenhänge mit segmental zugeordneten somatosensorischen und vegetativen Verschaltungen [16, 18, 20, 21] sowie die biomechanischen Verbindungen im Sinne des Tensegrity-Models [15] erklären komplexe Symptomkonstellationen und die teilweise weitreichenden Therapieerfolge, insbesondere dann, wenn vermeintlich „fern“ eines Symptoms therapiert wird.

Jeder Patient ist mit diesen komplexen Mechanismen in seiner Befundkonstellation hochgradig individuell, was eine strukturierte Aufarbeitung mit prospektiven randomisierten Studien hinsichtlich eines isolierten Symptoms erheblich erschwert. Weitere Studien zur Wirksamkeit manueller Therapieverfahren sollten nicht die alleinige Frage stellen, ob eine Symptomatik gebessert wird, sondern immer auch, ob eine segmentale und somatische Dysfunktion vorhanden ist, ob deren spezifische Therapie die Dysfunktion auflöst und daran geknüpft, ob die Symptomatik sich bessert.

## Fazit für die Praxis

- Manuelle Medizin basiert auf Neurophysiologie und Biomechanik.
- Manualmedizinische Therapie erfolgt über Setzen propriozeptiver Reize, die über A‑Beta-Fasern GABAerg (*GABA* Gamma-Aminobuttersäure) im Rückenmarkhinterhorn vermittelt werden.
- Segmentale, vegetative und biomechanische Aspekte können zu komplexen Symptomkonstellationen führen.
- Segmentale, vegetative und biomechanische Aspekte erlauben therapeutischen Einfluss auch scheinbar fern einer Symptomatik.
- Manuelle Medizin ist hilfreich in der Differenzialdiagnostik.
- Manuelle Medizin behandelt grundsätzlich die segmentale und somatische Dysfunktion und *nicht* ein Symptom.

## Abkürzungen

AV: Atrioventrikulär
BWK: Brustwirbelkörper
BWS: Brustwirbelsäule
ESSOMM: European Scientific Society of Manual Medicine
GABA: Gamma-Aminobuttersäure
HWK: Halswirbelkörper
ICD: International Statistical Classification of Diseases and Related Health Problems
LWK: Lendenwirbelkörper
LWS: Lendenwirbelsäule
MIP: Mobility, Irritation, Provocation
OSG: Oberes Sprunggelenk
RM: Rückenmark
ROM: „Range of motion“
ZNS: Zentralnervensystem
